# Inductive Effects on Intramolecular Hydrogen Bond Strength: An Investigation of the Effect of an Electron-Withdrawing CF_3_ Group Adjacent to an Alcohol Hydrogen Bond Donor

**DOI:** 10.1021/acs.jpca.3c03485

**Published:** 2023-09-15

**Authors:** Kaili Yap, Kristin D. Krantzman, Richard J. Lavrich

**Affiliations:** Department of Chemistry and Biochemistry, College of Charleston, 66 George Street, Charleston, South Carolina 29424, United States

## Abstract

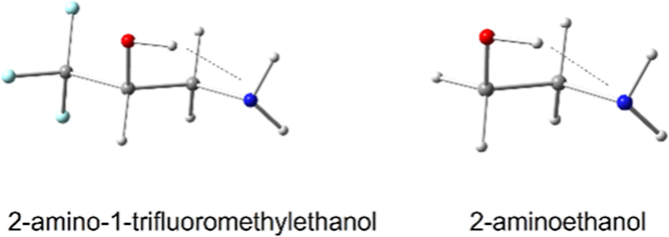

This combined experimental and theoretical study seeks to determine the role that inductive effects have on hydrogen bonds by an investigation into the change in intramolecular hydrogen bond strength in 2-amino-1-trifluoromethylethanol (2ATFME) relative to that in 2-aminoethanol (2AE). Toward this end, the rotational spectra of the normal, ^13^C, and ^15^N isotopologues have been measured using Fourier transform microwave spectroscopy and fit to the rotational, quadrupole coupling, and centrifugal distortion constants of the Watson A-reduction Hamiltonian. Structural parameters used to characterize the strength of the intramolecular hydrogen bond have been determined from the experimental structures of both 2ATFME and 2AE as well as from MP2/6-311++G(d,p) calculations. A comparison of these parameters in 2ATFME with those of 2AE indicates that the electron-withdrawing trifluoromethyl CF_3_ group strengthens the hydrogen bond. These include a 4% decrease in the distance between the donor and acceptor heavy atoms of the hydrogen bond, a 6% increase toward linearity of the OH···N angle, and a 23% decrease of the COH···N torsional angle toward planarity in 2ATFME relative to 2AE. This trend toward increased intramolecular hydrogen bond strength in 2ATFME is also observed within the ab initio structures.

## 1 Introduction

The conformations of linear amino alcohol monomers are stabilized by intramolecular hydrogen bonding between the alcohol and amine groups. The ability of these groups to act as both hydrogen bond donor (D–H) and acceptor (A) results in two hydrogen bonding motifs: (i) alcohol-to-amine (OH···N) and (ii) amine-to-alcohol (NH···O). The amine-to-alcohol intramolecular hydrogen bonding motif has been observed in the monomer subunit within aggregates of small linear amino alcohols in condensed phase studies^1,2^ as well as isolated gas phase amino alcohol monomers containing sterically hindering ring substituents.^3,4^ Studies of isolated, gas phase monomers of 2-aminoethanol (2AE),^5–8^ 3-aminopropanol (3AP),^8–12^ 4-aminobutanol,^13^ and 5-aminopentanol,^14^ however, have shown the preference for the formation of OH···N intramolecular hydrogen bonds.

Several geometric parameters can be used to characterize the strength of the intramolecular hydrogen bond.^15^ An increase in intramolecular hydrogen bond strength is associated with an increase in the covalent bond distance between the donor and hydrogen atoms, *r*(D–H), a decreased distance between the proton of the donor and the hydrogen bond acceptor, *r*(H···A), and a decrease in the separation between the donor and acceptor atoms, *r*(D···A). In addition, an increase in hydrogen bond strength is associated with a preferred donor directionality, with the angle between the atoms involved in the hydrogen bond θ (D–H···A) approaching linearity and a more planar torsional angle τ(CDH···A). The ability to adopt these geometric preferences may be hindered, however, by other factors such as the location of the molecular dipole, cooperative effects of multiple hydrogen bonding interactions, and steric effects within the molecule.

Inductive effects, from the presence of electron-withdrawing/donating groups near the donor and acceptor atoms, play a role in the strength of the intramolecular hydrogen bond. Considering the OH···N intramolecular hydrogen bond observed experimentally for linear amino alcohols, the following can be said about donor O and acceptor N. In general,^16^ an increase in the strength of the hydrogen bond results from either the addition of electron-withdrawing groups near the oxygen donor (due to an increase in the acidity of the proton) or the placement of electron donor groups near the nitrogen acceptor (causing a reduction of the s character of the nitrogen lone pair orbital and less tightly held electrons). The opposite is true for a weakening of the hydrogen bond; electron-donating groups near the oxygen donor and electron-withdrawing groups near the nitrogen acceptor.

Studies lending support to the role of inductive effects on the strength of the OH···N bond in amino alcohols have recently been reported. A microwave study^17^ of *N*-methyl-2AE observed two experimental conformers, both of which contain an OH···N intramolecular hydrogen bond. Upon addition of the electron-donating CH_3_ group near the nitrogen acceptor for both conformers, a modest decrease in the *r*(OH···N) bond distance (0.8 and 3.0%) and a modest increase of the θ(O–H···N) angle toward linearity (0.4 and 2.1%) relative to 2AE were observed, suggesting a strengthening of the intramolecular hydrogen bond. A joint spectroscopic [Fourier transform infrared and nuclear magnetic resonance (NMR)] and computational (density functional theory and molecular dynamics) study^18^ of *N*-methyl and *N*,*N*-dimethyl 3AP reports a systematic enhancement of the OH···N hydrogen bond with each methyl substitution. Recent high-level G4 ab initio calculations^19^ lend support to the above inductive effects by examining a series of linear amino alcohols having electron-withdrawing halogens placed either at the carbon atom adjacent to the donor or acceptor in the OH···N intramolecular hydrogen bond.

**Figure 1:**
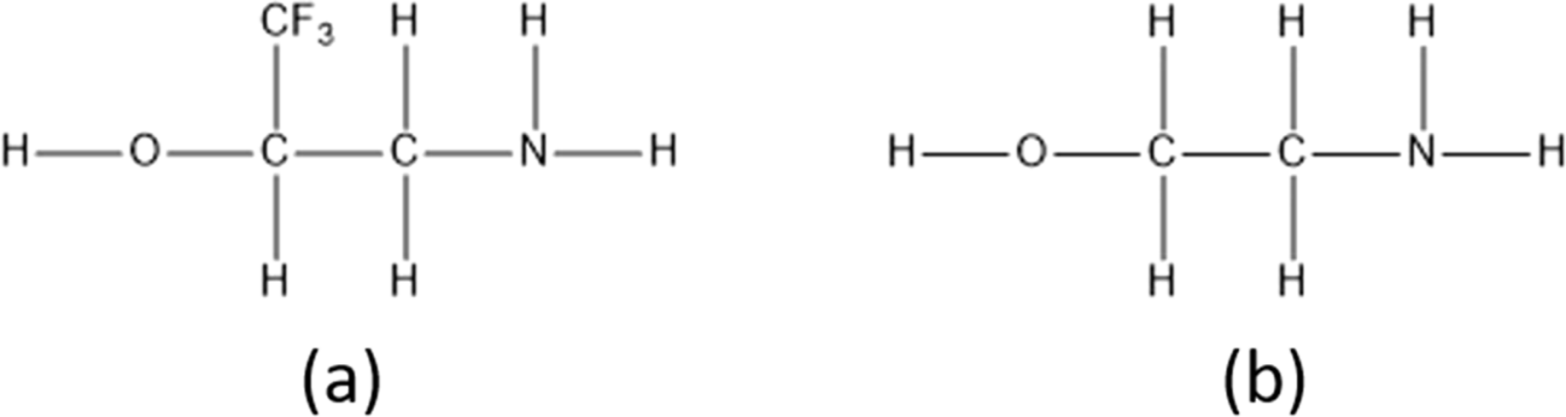
Addition of an electron-withdrawing CF_3_ group on the carbon adjacent to the alcohol donor of the OH···N intramolecular hydrogen bond in 2AE (b) yields 2ATFME (a).

The present study examines the effect of the addition of an electron-withdrawing CF_3_ group bonded to the carbon adjacent to the hydrogen bond donor oxygen. An investigation into the change in the strength of the intramolecular hydrogen bond in 2-amino-1-trifluoromethylethanol (2ATFME) relative to 2AE, shown in Figure 1, is undertaken.

High-resolution microwave spectra will be collected and analyzed along with ab initio methods to obtain highly precise structural information on the isolated, low-temperature amino alcohols. Such information may provide evidence of inductive effects on the intramolecular hydrogen bond strength. The addition of an electron-withdrawing CF_3_ group neighboring the donor group is expected to make the intramolecular hydrogen bond in 2ATFME stronger relative to 2AE.

## 2 Experimental Section

2ATFME was synthesized by treating 2-(trifluoromethyl)oxirane (purchased from Synquest Laboratories) dissolved in methanol with 7 N ammonia in methanol. Removal of solvent yielded nearly 100% yield of pure product (confirmed by NMR).

Rotational spectra were measured from 11 to 18 GHz using a pulsed molecular beam, a Fabry–Perot cavity spectrometer described in detail elsewhere.^20,21^ Briefly, the sample is seeded into an argon carrier gas at backing pressures of ∼1.5 atm. The heated reservoir nozzle, set to 90 °C, is oriented parallel to the cavity axis, resulting in rotational line widths on the order of 15 kHz (full width at half maximum) with line centers accurate to less than 2 kHz. The rotational temperature in expansion under these conditions is ∼2 K.

Short survey scans (on the order of 100–500 MHz) were collected for 2ATFME in order that signal intensity could be monitored. For each survey scan, 20 free induction decays were averaged and Fourier transformed at stepped frequency intervals of 0.5 MHz. Assigned transitions were later remeasured with additional averaging as necessary in order to fully resolve the hyperfine structure resulting from the quadrupolar nitrogen nucleus as well as the signal from the ^13^C and ^15^N isotopologues. The graphical user interface JB95^22,23^ was used to patch together short survey scans and individual measurements and to assign rotational quantum numbers to the energy levels involved in the rotational transitions.

## 3 Results

2ATFME exhibited a strong a- and b-type spectrum with c-type transitions roughly one-third of the intensity. Ninety-eight nuclear quadrupole hyperfine components from 29 rotational transitions (17 a-type, 9 b-type, and 3 c-type) were measured for the most abundant isotopologue of 2ATFME. Some strong transitions had unresolvable hyperfine structures and were not included in the fit. The hyperfine transition frequencies are available as Supporting Information (Table S1). The result of a global fit of rotational, centrifugal distortion, and nuclear quadrupole hyperfine coupling constants performed with Pickett’s SPFIT^24^ program with a Watson A-reduction Hamiltonian^25^ using the I^r^ representation is given in Table 1.

**Table 1:** Spectroscopic Constants of the Normal, ^13^C, and ^15^N Isotopologues of 2ATFME

	normal	^13^C-1	^13^C-2	^13^C-3	^15^N
A/MHz	3006.8831(2)^a^	2994.199(1)	2992.666(2)	3006.965(1)	2997.0405(1)
B/MHz	1664.5799(1)	1641.3506(2)	1628.5055(1)	1641.1545(1)	1616.3504(7)
C/MHz	1452.4610(1)	1450.7315(1)	1449.7566(2)	1449.7566(2)	1430.3838(9)
Δ_J_/kHz	0.219(2)	0.219^b^	0.219^b^	0.219^b^	0.219^b^
Δ_JK_/kHz	0.016(6)	0.016^b^	0.016^b^	0.016^b^	0.016^b^
Δ_K_/kHz	0.43(3)	0.43^b^	0.43^b^	0.43^b^	0.43^b^
δ_J_/kHz	–0.035(1)	–0.035^b^	–0.035^b^	–0.035^b^	–0.035^b^
δ_K_/kHz	0.004(1)	0.004^b^	0.004^b^	0.004^b^	0.004^b^
χ_aa_^c^/MHz	1.918(2)	1.92(2)	1.91(2)	1.91(2)	
χ_bb_^c^/MHz	–3.5062(7)	–3.466(4)	3.550(4)	–3.508(4)	
σ^d^/kHz	1.8	1.7	2.3	2.3	3.4
N^e^	98	30	29	29	9

a Error in parentheses are in units of units of the last digit.

b Values fixed at those found for the normal isotope.

c Quadrupole coupling constants determined by fitting to 3/2χaa and 1/4(χbb – χcc) and using the traceless nature of the quadrupole coupling tensor.

d Root-mean-square error of the fit.

e Number of lines in the fit.

The stronger transitions of a smaller set of a- and b-type transitions were measured for each of the ^13^C and ^15^N isotopologues in natural abundance. Centrifugal distortion constants were fixed at values determined for the normal isotopologue during fits of the rotational and nuclear quadrupole coupling constants for each of the ^13^C isotopologues and for the rotational constants of the ^15^N isotopologue (see Table 1). Transition frequencies for the ^13^C (Table S2) and ^15^N isotopologues (Table S3) are available in the Supporting Information. Despite extensive searching, no signal from the additional conformers was found.

## 4 Computational

**Figure 2:**
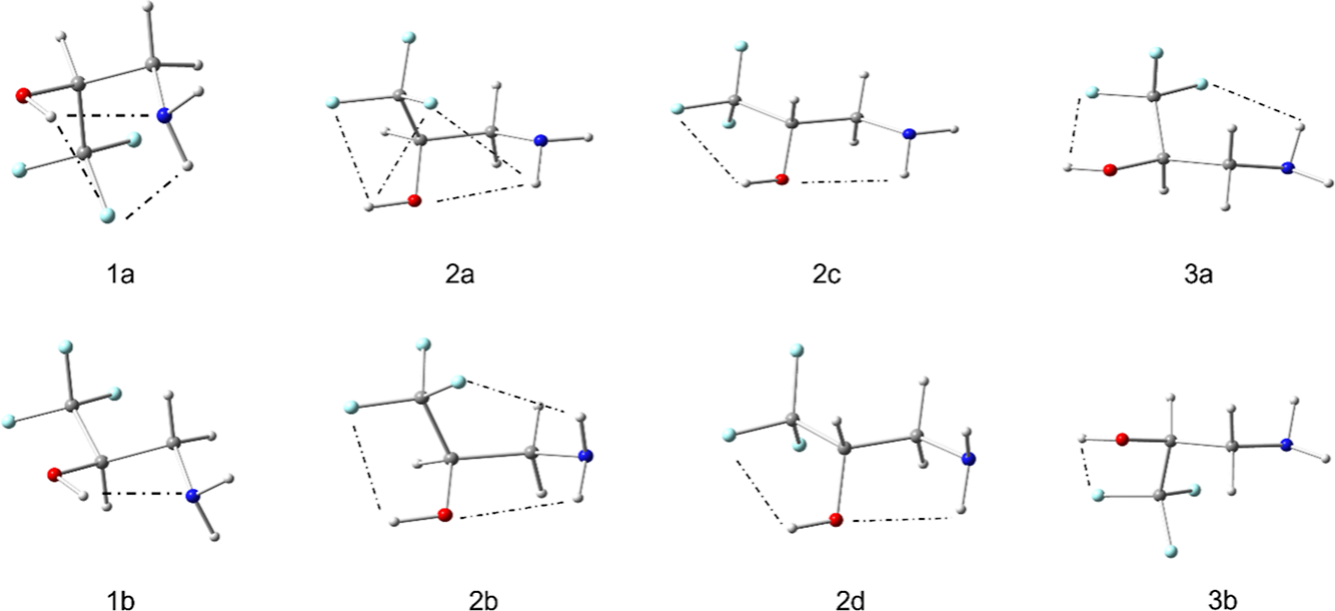
Conformers of 2ATFME from MP2/6-311++G(d,p) calculations.

Quantum calculations were performed at the MP2/6-311G++(d,p) level using the Gaussian 09 suite of programs.^26^ The resulting equilibrium conformations from the geometry optimizations are shown in Figure 2 and briefly described in Table 2. Single-point calculations were carried out to determine dipole moment components, nuclear quadrupole coupling constants, and rotational constants of the ^13^C and ^15^N isotopologues for each of the conformational minima.

**Table 2:** Calculated MP2/6-311++G(d,p) Energetic and Spectroscopic Data of Low Energy Conformers of 2ATFME

	1a	1b	2a	2b	2c	2d	3a	3b
Δ*E*^a^	0	5.3	18.7	13.1	9.1	11.4	16.6	22.6
A/MHz	3008.209	3401.083	2882.240	2931.942	3307.363	3298.423	2670.287	2638.351
B/MHz	1648.983	1418.007	1693.999	1646.522	1421.043	1410.402	1784.632	1818.541
C/MHz	1457.303	1250.349	1463.153	1434.2356	1242.341	1234.704	1363.514	1377.309
|μ_a_| (D)	3.0	3.9	1.6	0.6	1.4	0.4	1.2	2.1
|μ_b_| (D)	2.3	2.4	1.2	1.4	0.2	1.0	0.3	1.0
|μ_c_| (D)	1.0	0.8	1.8	1.1	0.1	2.1	0.1	1.4
χ_aa_ (MHz)	2.09	2.76	–1.24	–2.47	2.93	0.38	2.17	–1.49
χ_bb_ (MHz)	–3.70	–4.58	–0.25	–0.03	1.83	–0.78	2.64	–0.53
τ_1_^b^/deg	43.3	51.8	–61.3	–49.4	–66.4	–62.2	165.8	–177.2
τ_2_^c^/deg	–80.1	171.7	61.7	73.1	172.7	176.7	–74.3	62.1
Δ*I*_rms_^d^ amu·Å^2^	3.2	174.2	26.5	14.0	175.5	183.6	88.2	94.5

a kJ/mol relative to the global minimum 1a.

b Backbone torsional angle (NC_2_C_1_O).

c Backbone torsional angle (NC_2_C_1_C_3_).

d Root-mean-square averages of the differences between observed and calculated moments of inertia, where Δ*I* = [*I*_*x*_(exp) – *I*_*x*_(calc)] and *x* = *a*, *b*, and *c* for each isotopologue.

As discussed above, amino alcohols have two intramolecular hydrogen bonding motifs (either alcohol-to-amine or amine-to-alcohol). The presence of a hydrogen bond accepting fluorine makes additional OH···F and NH···F hydrogen bonds possible. Calculated minima are assigned to three general classes; class 1 contains an OH···N hydrogen bond, class 2 contains an NH···O hydrogen bond, and class 3 has no hydrogen bonding between the alcohol and amine groups. Within each class are conformers containing additional intramolecular hydrogen bonds with fluorine.

The two lowest-energy conformations from the calculations belong to class 1 and are stabilized by an OH···N intramolecular hydrogen bond (conformers 1a and 1b). This is consistent with previous microwave studies of amino alcohols.^9,11,13,14^ The global minimum conformation, 1a, has the alcohol group involved in a three-centered hydrogen bond (the alcohol proton having a bifurcated interaction with nitrogen and fluorine acting as acceptors). In addition, the amino nitrogen is involved in a two-centered hydrogen bond, donating a proton to the same fluorine as the oxygen does. For conformer 1b, there are no additional hydrogen bonds other than the two-centered alcohol-amine hydrogen bond.

Two sets of higher-energy 2ATFME conformers were found; those that contain an amine-to-hydroxyl intramolecular hydrogen bond (class 2) and those that contain no hydrogen bonding interaction between the amine and alcohol groups (class 3). All of the conformers in class 2 have the alcohol involved in at least one hydrogen bond with fluorine. Conformer 2a has both the alcohol and amino groups involved in three-centered hydrogen bonds, while the other three (2b–2d) each contain two-centered OH and NH hydrogen bonds. Within class 2, one of the four structures, 2b, has both amino protons involved in two-centered hydrogen bonds (one forming NH···O and the other NH···F). Both high energy conformers found to belong to class 3 (3a and 3b) have a two-centered hydrogen bond involving O and F but with 3a having an additional two-centered hydrogen bond involving N and F.

## 5 Discussion

In order to correlate the experimental rotational spectrum with a particular ab initio conformer, the calculated spectroscopic parameters (rotational constants, dipole moment components, and nuclear quadrupole coupling constants) can be used to eliminate theoretical structures. Rotational constants are compared by first converting to moments of inertia and then calculating Δ*I*_rms_, the root-mean-square (RMS) average of the differences between observed and calculated moments of inertia about the three principal axes (*a*, *b*, and *c*) for all isotopologues. Referring to these parameters given in Table 2, the global minimum ab initio conformation 1a is the only conformer consistent with experiment.

Evidence supporting the elimination of all ab initio conformers other than 1a is provided by the relative strengths of the observed rotational transitions, which are governed by the magnitude of the components of the dipole moment along the principal axes. The observed spectrum of 2ATFME contains stronger a- and b-type transitions with weaker c-type transitions at roughly one-third of the intensity. Ab initio conformers 2a, 2b, 2d, and 3b all predict strong c-type transitions, 2c and 3a both predict weak b-types, while 2b and 2d are calculated to have a weak a-type spectrum.

The calculated value of Δ*I*_rms_, given in Table 2, for all conformers other than the global minimum 1a confirms that they are not consistent with the experiment. While the second conformer having the preferred OH···N intramolecular hydrogen bonding motif, 1b, has a calculated relative energy close to the global minimum and dipole moment components consistent with experiment, its Δ*I*_rms_ value of 174.2 amu Å^2^ removes it from contention. Conformers containing a NH···O intramolecular hydrogen bond (2a–2d) have Δ*I*_rms_ values ranging from 14.0 to 183.6 amu Å^2^. For the conformers with no hydrogen bond between the amino and alcohol groups, Δ*I*_rms_ values are on the order of 100 amu Å^2^. Conformer 1a, having a Δ*I*_rms_ value 1-2 orders of magnitude less than all other conformers, best correlates with the experiment.

Finally, inspection of the calculated nuclear quadrupole coupling constants shows only conformers 1a and 1b to be consistent with experiment (although both χ_aa_ and χ_bb_ for conformer 1b are calculated to be 30–40% too high). All other ab initio conformers have either inconsistent signs or magnitudes in excess of double those of the experimental values. Based on consideration of the above, the experimental spectra can therefore be assigned to ab initio conformer 1a.

The absence of conformers other than 1a in the experimental spectra is further suggested by energy considerations. Using the calculated relative energies of the conformers of 2ATFME, along with the temperature of the heated reservoir nozzle (∼363 K), the Boltzmann distribution was used to provide an estimate of the pre-expansion populations of each. It is estimated that prior to expansion, conformers 1a and 1b make up 93% of the population (79% 1a and 14% 1b).

Conformational relaxation^27^ during the supersonic expansion is expected if the interconversion barrier is smaller than 2 kT, which at the pre-expansion temperature of the present study is 504 cm^–1^. Conformer 1b, the only other conformer that is expected to have an appreciable population prior to expansion, is calculated to lie 443 cm^–1^ above the global minimum and is expected to be absent from the jet.

The moments of inertia for each isotopologue were used to determine a least-squares fit^28^ structure. The global minimum ab initio conformation of 2ATFME, 1a, was used as input for the least-squares fit, which adjusted internal coordinates describing the positions of heavy atoms. The positions of the hydrogens were fixed at ab initio values. In addition, the atomic coordinates of the carbon and nitrogen atoms were calculated from the isotopologue moments of inertia using Kraitchman’s equations for single isotopic substitution.^29,30^

A comparison of the least-squares fit parameters with those in the starting ab initio structure is given in Table 3. The fit converged after changing the structure of 1a only slightly; fitted bond lengths are within a few hundredths of an angstrom at most of those from the ab initio global minimum, while fitted bond and torsional angles changed by 1% or less. The resulting least-squares fit structure reproduces the experimental moments of inertia well with Δ*I*_rms_ = 0.014 amu Å^2^.

**Table 3:** Heavy Atom Bond Lengths, Angles, and Torsional Angles from the Least-Squares Fit and Global Minimum Ab Initio Structures of 2ATFME

	least-squares^a^	1a
N–C_2_/Å	1.461(3)	1.467
C_1_–C_2_/Å	1.55(1)	1.536
C_1_–C_3_/Å	1.515(7)	1.524
C_1_–C_2_–N/degrees	109.2(3)	108.52
C_2_–C_1_–O/degrees	111.8(4)	110.52
N–C_2_–C_1_–O/degrees	–43.4(5)	–43.27
N–C_2_–C_1_–C_3_/degrees	80.5(4)	80.14

a With 1a as the starting structure.

Table 4 gives the heavy atom atomic coordinates for which spectroscopic isotopologue data has been measured from the least-squares fit and global minimum ab initio structure. The Kraitchman atomic coordinates are also listed in Table 4. Comparison of the Kraitchman coordinates and those resulting from the least-squares fit shows that they are in excellent agreement.

**Table 4:** Atomic Coordinates (Å) of 2ATFME Determined from the Global Minimum Ab Initio Structure, Least-Squares Fit Structure, and Kraitchman’s Equations

		Ab initio 1a	least-squares fit^a^	Kraitchman^b^
C(1)	*a*	0.413	0.40(1)	0.392(4)
	*b*	0.513	0.516(6)	0.510(3)
	*c*	–0.676	–0.672(5)	0.675(2)
C(2)	*a*	1.571	1.576(3)	1.573(1)
	*b*	–0.493	–0.501(8)	0.493(3)
	*c*	–0.759	–0.753(4)	0.756(2)
C(3)	*a*	–0.814	–0.815(2)	0.806(2)
	*b*	–0.089	–0.091(3)	0.04(3)
	*c*	–0.004	–0.003(2)	0.08i(2)
N	*a*	2.253	2.263(2)	2.265(1)
	*b*	–0.541	0.536(5)	0.538(3)
	*c*	0.539	0.537(1)	0.536(3)

a With 1a as the starting structure.

b Absolute values of coordinates determined.

Table 5 compares the parameters used to characterize hydrogen bond strength for both the global minimum ab initio as well as the experimental structures of 2ATFME and 2AE. The least-squares fit structure of 2AE was obtained using rotational constants from the ^13^C, ^18^O, and ^15^N isotopologues from previous studies.^4–8^

**Table 5:** Structural Parameters Describing the Intramolecular Hydrogen Bond in the Ab Initio and Experimental Structures of 2ATFME and 2AE

	Ab initio			Least-squares fit		
parameter	2ATFME	2AE	% change^a^	2ATFME	2AE	% change^a^
*r*(O–H)/Å	0.973	0.965	0.8	0.973^b^	0.965^b^	0.8
*r*(OH···N)/Å	2.048	2.233	–8.3	2.059	2.266	–9.1
*r*(O···N)/Å	2.695	2.796	–3.6	2.706	2.820	–4.0
θ(O–H···N)/deg	122.09	116.18	5.1	122.26	115.51	5.8
τ(COH···N)/deg	11.83	16.22	–27.1	12.12	15.77	–23.1

a Relative to 2AE.

b Fixed at ab initio value.

Structural evidence to support the expected trend of increasing hydrogen bond strength from the inductive effect of electron-withdrawing substituents on the carbon adjacent to the alcohol group is observed in the ab initio structures. Upon addition of the electron-withdrawing CF_3_ group, a 0.8% increase in the donor O–H bond length, an 8.3% decrease in the donor–acceptor OH···N distance, a widening of the θ(O–H···N) angle, and a significantly more planar τ(COH···N) are all indicative of a stronger intramolecular hydrogen bond.

The trends observed for the ab initio calculations are preserved in the experimental structures. The effect of the electron-withdrawing CF_3_ group adjacent to the hydrogen bond-donating alcohol group decreases the hydrogen bond distance between the alcohol proton and the nitrogen acceptor by 9%. Concomitant with this is a 4% decrease in the distance between the oxygen and nitrogen atoms involved in the hydrogen bond. In addition, a 6% increase toward linearity of the OH···N bond angle (from 115.51° in 2AE to 122.26° in 2ATFME) and a 23% decrease toward planarity (15.77° in 2AE to 12.13° in 2ATFME) indicate an increase in the strength of the hydrogen bond.

## 6 Conclusions

Inductive effects on intramolecular hydrogen bond strength have been examined by substitution of one of the hydrogens on the CH_2_ group adjacent to the hydrogen bond donor alcohol in 2AE with an electron-withdrawing CF_3_ group. It was found that due to the increase in acidity of the alcohol proton from the inductive electron-withdrawing effect, a stronger intramolecular hydrogen bond was observed. Present studies are underway to examine the effect of substitution with an electron-donating CH_3_ group as well as probing the strength of the intramolecular hydrogen bond in 2ATFME by introducing competing intermolecular hydrogen bonds by complexation with H_2_O.

## Supporting Information Available

Frequencies of the assigned nuclear quadrupole hyperfine transitions of the normal isotopologue of 2ATFME (PDF)
Frequencies of the assigned nuclear quadrupole hyperfine transitions of the ^13^C isotopologues of 2ATFME (PDF)
Rotational transition frequencies of the ^15^N isotopologue of 2ATFME (PDF)
