# Supplementary material for: Inductive Effects on Intramolecular Hydrogen Bond Strength: An Investigation of the Effect of an Electron-Withdrawing CF3 Group Adjacent to an Alcohol Hydrogen Bond Donor
Source: J Phys Chem A. 2023 Sep 15;127(38):7892–7. doi: 10.1021/acs.jpca.3c03485 (PMC10544021; doi:10.1021/acs.jpca.3c03485)
Supplement: Supplementary file 3 — jp3c03485_si_003.pdf [file jp3c03485_si_003.pdf]

Inductive Effects on Intramolecular Hydrogen Bond Strength: An Investigation of the Effect of an Electron Withdrawing CF3 Group Adjacent to an Alcohol Hydrogen Bond Donor

Kaili Yap, Kristin D. Krantzman, Richard J. Lavrich

Department of Chemistry and Biochemistry, College of Charleston, 66 George St., Charleston, SC., 29424

Table S3. Rotational Transition Frequencies of the ^{15}N Isotopologue of 2-amino-1-trifluoromethylethanol

J'	K'_p	K'_o	J''	K''_p	K''_o	ν (MHz)	Δν (kHz)
6	0	6	5	0	5	17786.863	2.1
3	3	1	2	2	0	16501.493	-2.6
5	1	4	4	1	3	15610.364	-4.5
5	0	5	4	0	4	14921.631	-2.6
5	0	5	4	1	4	14166.935	2.8
3	2	2	2	1	1	13282.258	0.0
4	1	3	3	1	2	12520.434	4.6
4	0	4	3	0	3	12020.073	-0.4
4	1	4	3	1	3	11782.767	-2.5
